# Efficacy and Safety of Direct Oral Anticoagulants in Elderly Patients With Atrial Fibrillation: A Network Meta-Analysis

**DOI:** 10.3389/fmed.2020.00107

**Published:** 2020-04-07

**Authors:** Kaisheng Deng, Jinqun Cheng, Shufang Rao, Huafu Xu, Lixia Li, Yanhui Gao

**Affiliations:** School of Public Health, Guangdong Pharmaceutical University, Guangzhou, China

**Keywords:** atrial fibrillation, antithrombotic, elderly patients, direct oral anticoagulants, network meta-analysis

## Abstract

**Background:** Direct oral anticoagulants (DOACs) have been widely used in patients with atrial fibrillation (AF) for antithrombotic prophylaxis, which were shown to have a favorable risk–benefit profile. However, there are no guidelines for the use of DOACs in elderly patients (aged ≥75 years) with AF, which creates uncertainty about the optimal antithrombotic treatment in these patients.

**Methods:** After comprehensively searching Embase, PubMed, and Cochrane databases, five phase III randomized controlled trials involving 28,137 elderly participants were included in this study. The efficacy outcome was stroke or systemic embolism, and the safety outcome was major bleeding. We conducted a network meta-analysis by using a Bayesian random-effect model for the first time to evaluate the efficacy and safety of main DOACs (apixaban, edoxaban, rivaroxaban, and dabigatran) and warfarin in elderly patients with AF. Hazard ratios (HRs) and their corresponding 95% confidence intervals (CIs) were used to assess the effect of drugs on efficacy and safety. The rank probabilities were used to reflect the hierarchy of drugs, and a larger rank probability value symbolized a better rank of drugs.

**Results:** In the prophylaxis of stroke or systemic embolism, apixaban was found to be the best among DOACs compared to warfarin (HR, 0.71; 95% CI: 0.33–1.50), though this finding was not statistically significant. Apixaban ranked the best (rank probabilities, 41.2%) in efficacy of drugs, followed by rivaroxaban, edoxaban, dabigatran, and warfarin (rank probabilities, 31.8, 15.9, 10.9, and 0.2%, respectively). In reducing the risk of major bleeding, apixaban was found to be the best among DOACs too, compared to warfarin (HR, 0.64; 95% CI: 0.33–1.30), though this finding was not statistically significant. In safety, apixaban ranked the best (rank probabilities, 71.4%), followed by edoxaban, dabigatran, warfarin, and rivaroxaban (rank probabilities, 21.0, 5.8, 0.9, and 0.8%, respectively).

**Conclusions:** DOACs showed a lower incidence of stroke/systemic embolism and major bleeding compared with warfarin in antithrombotic therapy in elderly patients (aged ≥75 years), with apixaban being the best of those interventions. Therefore, apixaban should be given priority as an anticoagulant in stroke prevention for elderly patients with AF.

## Introduction

Atrial fibrillation (AF) is one of the most common cardiovascular diseases worldwide. AF is associated with a fivefold increase in stroke risk, and one in five cases of stroke is attributed to this arrhythmia (1). AF is not only associated with an increased risk of stroke but also increases the risk of heart failure and all-cause mortality (2). AF has also been associated with silent brain lesions, as well as cognitive impairment and dementia (3, 4). Once AF is diagnosed, anticoagulation therapy is initiated in most patients to prevent stroke and other thromboembolic events, thereby significantly lowering morbidity and mortality (5).

The prevalence of AF increases with age, and two-thirds of patients with AF are the elderly (aged ≥75 years) (6). The Framingham Heart Study group had shown age to be the greatest risk factor for AF, surpassing other risk factors, including male sex, obesity, diabetes mellitus, smoking, hypertension, heart failure, and coronary artery disease (7). The Multi-Ethnic Study of Atherosclerosis (MESA study) also reported age-specific incidence rates of AF in individuals aged 65–74 and 75–84 years of 3.4 and 8.6% for Chinese, 4.9 and 10.6% for non-Hispanic Blacks, 7.3 and 9.4% for Hispanics, and 13.4 and 19.6% for non-Hispanic Whites, respectively, showing that the incidence rate of AF is higher for the elderly in many races (8). There are poorer quality of life, larger number of hospitalizations, and more cardiovascular events in elderly patients with AF, compared to patients younger than 75 years old (9). In addition, older age is a known factor that can increase the risk of stroke/systemic embolism, which is one of the most common complications of AF; thus, the occurrence of comorbidities is frequent in the elderly (6).

Antithrombotic prophylaxis is crucial for patients with AF to prevent them from incurring comorbidities, improve quality of life, and reduce death. Since 2009, direct oral anticoagulants (DOACs) have been widely used in patients with AF for prevention of stroke or systemic embolism (10). Many studies have repeatedly proved that the main DOACs (apixaban, edoxaban, rivaroxaban, and dabigatran) have better efficacy than warfarin (one of the traditional drugs for stroke prevention in patients with AF) in antithrombotic therapy (10–13). These drugs can inhibit thrombin directly or activated factor X(Xa), exhibit fewer drug interactions (avoiding the need for strict diet control), and have rapid onset of action compared to warfarin (14).

However, despite extraordinarily high stroke risk, the elderly have been paradoxically less likely to receive oral anticoagulation therapy (6). Compared with young patients, the elderly suffered higher risk of bleeding in anticoagulant therapy. Bleeding is one of the most common side effects of patients with AF in anticoagulant therapy (1). The fear of bleeding may explain the underuse of oral anticoagulation in the elderly (15). When there was no significant difference between the efficacy of DOACs in the elderly and in the young (16), the assessment of safe drugs is more necessary for the elderly patients. After a 10-year development of the use of DOACs, a number of systematic reviews and meta-analyses have conducted detailed studies on the main DOACs in patients with AF for evaluating their efficacy and safety and indicated that apixaban offered the most favorable efficacy and safety profile in patients with AF of all ages (17–19).

However, up to now, there are no recommendations and guidelines for the use of DOACs specifically for the elderly. Whether apixaban is the best drug for elderly patients with AF is still unknown. The aim of the current study was exactly to provide suggestions for the use of DOACs in elderly patients and help clinicians to find the best choice depending on the individual conditions of the elderly patients and to maximize the benefits from the drugs for stroke/systemic embolism prevention while minimizing the risk of major bleeding.

In this study, we conducted a network meta-analysis by using a Bayesian random-effect model for the first time to assess the efficacy and safety of DOACs in elderly patients with AF. Network meta-analysis can evaluate multiple therapeutic strategies simultaneously and rank treatments based on efficacy and safety (20), to provide reference for clinicians in choosing the best treatment for patients.

## Materials and Methods

### Data Sources and Search Strategy

PubMed, EMBASE, and Cochrane databases were comprehensively searched using a particular strategy up to August 2019. Two reviewers (K. S. Deng and J. Q. Cheng) independently performed this search for the main oral anticoagulant drugs (warfarin, apixaban, edoxaban, rivaroxaban, and dabigatran). We searched studies by using keywords that included “Atrial fibrillation” or “AF” or “non-valvular AF” and “elderly patients” or “advanced age” or “older age” and “anticoagulation” or “antithrombotic” or “anticoagulants” or “warfarin” or “dabigatran” or “apixaban” or “rivaroxaban” or “edoxaban.” The electronic search strategies were provided in the online Supplementary S1. We also reviewed similar articles and the corresponding reference list of each retrieved study to identify any relevant studies that may have been neglected. We also searched for gray and unfinished studies to make the search more comprehensive. The Preferred Reporting Items of Systematic Reviews and Meta-analysis (PRISMA) diagram showed the search for the selection of references (21).

### Selection Criteria

Two authors (K. S. Deng and J. Q. Cheng) independently screened the title and abstract of each identified article and then reviewed the full text of each article according to the following inclusion and exclusion criteria. Any disagreements or uncertainties between the reviewers were resolved by consensus, and the final decision was made by discussion with the third co-author (S. F. Rao).

The inclusion criteria of the studies were as follows: (a) reported efficacy and safety outcomes by age subgroups (aged ≥75 and <75 years), (b) was a phase III randomized controlled trial (RCT) of a treatment group (DOACs) and a control group (warfarin), and (c) had outcomes of “stroke or systemic embolism” and “major bleeding.”

The exclusion criteria were as follows: (a) articles that repeated already included RCTs, such as systematic reviews, meta-analyses, and conference abstracts; (b) studies without a definition of endpoints or had endpoints that do not relate to AF; and (c) studies that do not contain information about the efficacy and safety of DOACs in specific subgroup patients with AF (aged ≥75 years).

In this study, the efficacy outcome was stroke or systemic embolism, and the safety outcome was major bleeding according to the definition of original researches (22, 23).

### Data Extraction

Two authors (K. S. Deng and J. Q. Cheng) extracted data independently in an electronic database. The extracted data included the first author's name, the year of publication, patient characteristics, the sample size of the population, treatment, control, follow-up duration, the outcomes of “stroke/systemic embolism” or “major bleeding,” and study design. If a trial had any uncertainties, the corresponding author of the publication was contacted to provide clarity or relevant information.

### Quality Assessment

Two authors (K. S. Deng and J. Q. Cheng) independently evaluated the quality of studies. The quality of these studies was assessed by the Cochrane risk-of-bias assessment because each of the study that we included in this research was an RCT. Because of the relatively large number of participants that each study involved and the rigorous inclusion and exclusion criteria, fewer than 10 studies were included.

### Statistical Analysis

The data were abstracted and analyzed by using a network meta-analysis method, which can combine direct and indirect evidence in a mixed-treatment comparison (20). Before conducting network meta-analysis, we performed conventional pairwise meta-analyses for DOACs that were directly compared in RCTs. We performed this network meta-analysis in a Bayesian random-effect model assuming a binomial likelihood and using “complementary log-log” as the link function. The network meta-analysis was conducted using the “gemtc” package, which recalls JAGS in R for Markov chain Monte Carlo (MCMC) sampling (24). Each model was fitted by setting 1,000 adapting iterations followed by 20,000 iterations (25). Convergence was checked using trace plots and the Brooks–Gelman–Rubin diagnostic (26). Outputs from the model were presented as hazard ratios (HRs) and their corresponding 95% confidence intervals (CIs). The rank probabilities were used to reflect the hierarchy of drugs, and a larger rank probability value symbolized a better rank of drugs. In addition, we used the “anohe” approach to access the heterogeneity of the current study. The publication bias of five included studies was assessed by funnel plots and Egger's test. Egger's test is a method for testing publication bias; when the *P* value of the result is > 0.05, it indicates that there is no obvious publication bias in the study (27). The evidence network plot for comparison of various treatment measures was drawn by using STATA (version 15.0, STATA MP). The stacked bar charts that can reflect the hierarchy of interventions were drawn by R software using the “ggplot2” package.

## Results

**Figure 1:**
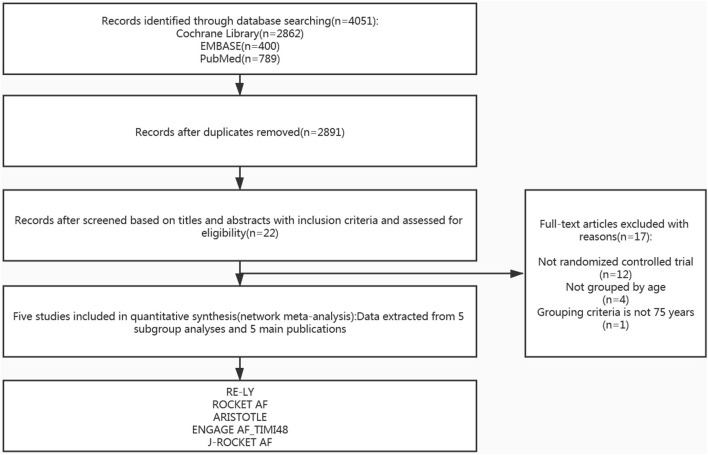
Study flow diagram of the literature search and selection process of the included studies.

**Figure 2:**
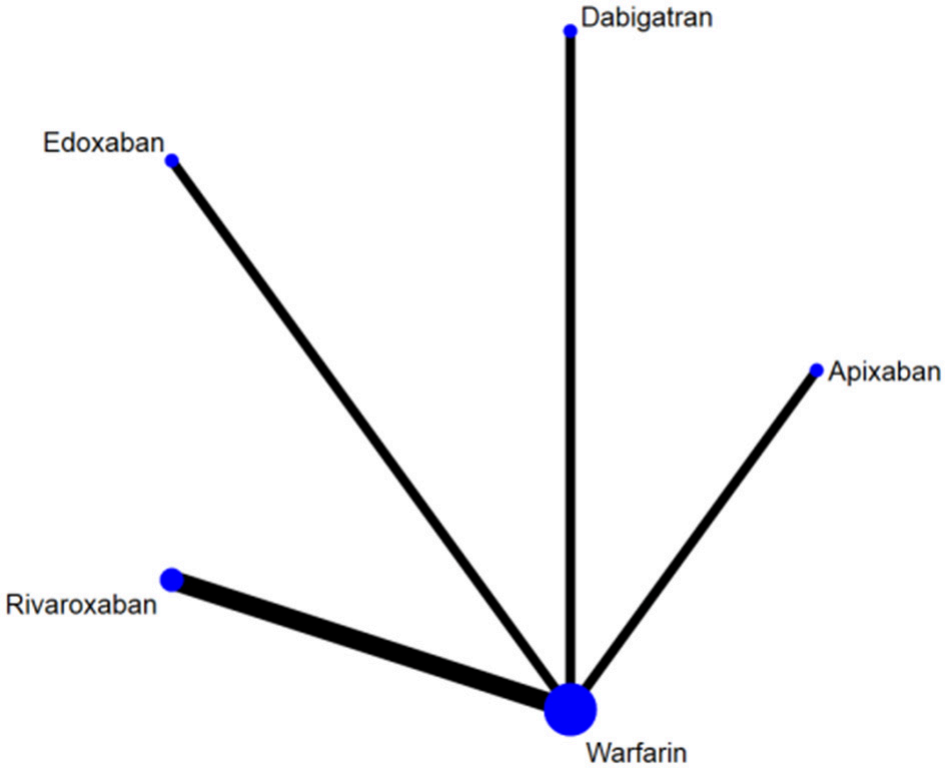
The evidence network plot of included RCTs. The size of the nodes is proportional to the number of patients randomized to receive the treatment. The width of the lines is proportional to the number of trials included in the study.

After screening and selecting, we finally included five phase III RCTs involving 28,137 elderly participants (28–32). The details of the searching and selecting process were illustrated in Figure 1. Four types of DOAC and warfarin interventions for elderly patients with AF published up to August 2019 were included. We did not find any gray study or unpublished trial associated with our research. Table 1 summarized the basic information and essential baseline characteristics of the trials. The quality of these trials was assessed using the Cochrane risk-of-bias assessment because they are all RCTs. The average score in the Cochrane risk analysis of five included RCTs was 7 (Supplementary S2), showing high-quality evidence to continue our study. The evidence network plot of the studies is shown in Figure 2. A summary of the results from the network meta-analyses on efficacy and safety is shown in Table 2.

**Table 1:** Summary of RCTs included in the network meta-analysis.

**Studies**	**Study design**	**Sample size** **(all/elderly)**	**Treatment** **(dosing regimen)**	**Control** **(dosing regimen)**	**Follow-up**	**Age, mean**	**Male, %**	**Number of patients lost to follow-up**	**Hazard ratios of stroke/systemic embolism,** **HR (95% CI)**	**Hazard ratios of major bleeding,** **HR (95% CI)**
ARISTOTLE, 2014	RCT	18,201/5,678	Apixaban (5 mg/bid)	Warfarin (INR^*^: 2.0–3.0)	1.9 years	79.0	46.2	1,496	0.71 (0.53–0.95)	0.64 (0.52–0.79)
ENGAGE AF-TIMI48, 2016	RCT	21,105/8,474	Edoxaban (60 mg/day)	Warfarin (INR: 2.0–3.0)	1.8 years	\	42.2	\	0.83 (0.66–1.04)	0.83 (0.70–0.99)
ROCKET AF, 2014	RCT	1,278/498	Rivaroxaban (20 mg/day)	Warfarin (INR: 2.0–3.0)	2.8 years	76.0	55.0	\	0.80 (0.63–1.02)	1.11 (0.92–1.34)
RE-LY, 2011	RCT	18,113/7,258	Dabigatran (110 mg/day)	Warfarin (INR: 2.0–3.0)	2.5 years	79.0	26.9	2,269	0.88 (0.66–1.17)	1.01 (0.83–1.23)
JROCKET, 2014	RCT	13,150/6,229	Rivaroxaban (20 mg/day)	Warfarin (INR: 2.0–3.0)	2.0 years	\	\	\	0.55 (0.22–1.40)	1.51 (0.68–3.32)

* *INR, International normalized ratio, indicator for monitoring warfarin dosage and efficacy*.

**Table 2:** Summary of the results from network meta-analyses on efficacy and safety outcomes (lower triangle, stroke/systemic embolism; upper triangle, major bleeding).

**HR 95% CI**	**Warfarin**	**Dabigatran**	**Apixaban**	**Rivaroxaban**	**Edoxaban**
Warfarin		1.00 (0.59, 1.70)	0.64 (0.33, 1.30)	1.20 (0.83, 1.90)	0.83 (0.48, 1.40)
Dabigatran	0.88 (0.44, 1.80)		0.64 (0.27, 1.50)	1.20 (0.63, 2.40)	0.82 (0.38, 1.80)
Apixaban	0.71 (0.33, 1.50)	0.81 (0.29, 2.30)		1.90 (0.89, 4.30)	1.30 (0.54, 1.30)
Rivaroxaban	0.73 (0.40, 1.20)	0.84 (0.32, 1.90)	1.00 (0.38, 2.50)		0.70 (0.33, 1.30)
Edoxaban	0.83 (0.41, 1.70)	0.94 (0.35, 2.50)	1.20 (0.41, 3.30)	1.10 (0.49, 2.90)	

*On the lower triangle, the column-defining treatment is compared to the row-defining treatment, and the hazard ratio (HR) <1 favors the column-defining treatment. On the upper triangle, the row-defining treatment is compared to the column-defining treatment, and (HR) <1 favors the row-defining treatment*.

**Figure 3:**
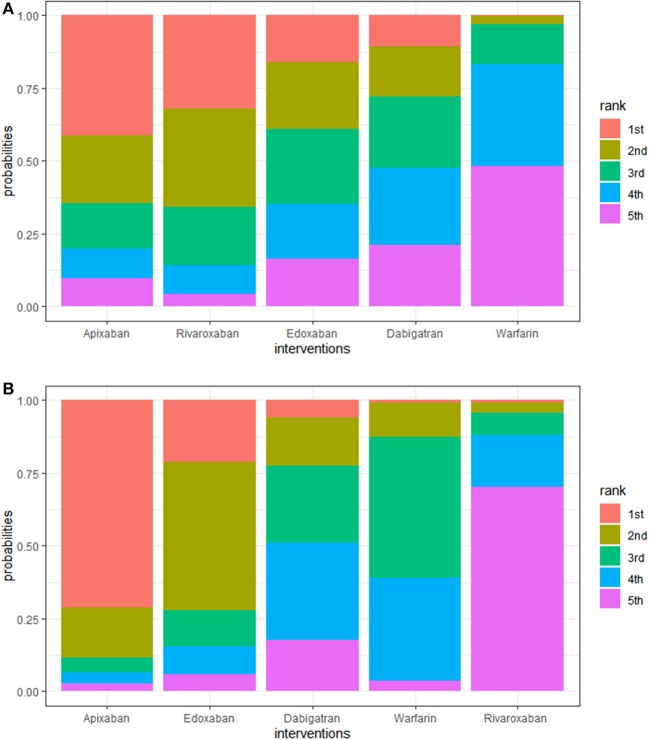
**(A)** Rank plot of interventions in efficacy. A larger width of the red column indicated that the drug is more likely to be the best in efficacy. Interventions are ranged from best to worst. For example, the ranking suggests that warfarin posed the worst efficacy in elderly patients with AF, while apixaban posed the best. **(B)** Rank plot of interventions in safety. A larger width of the red column indicated that the drug is more likely to be the best in safety. Interventions are ranged from best to worst.

In the prophylaxis of stroke or systemic embolism, apixaban was the best among the DOACs compared to warfarin (HR, 0.71; 95% CI: 0.33–1.50), though this result was not statistically significant. In reducing the risk of major bleeding, apixaban was the best among DOACs too, compared to warfarin (HR, 0.64; 95% CI: 0.33–1.30), though this finding was also not statistically significant. Additionally, the rank probabilities of each drugs were shown in Table 3, which could reflect the probable hierarchy of each drug on efficacy and safety. In the prevention of stroke or systemic embolism, apixaban ranked the best (first-rank probability, 41.2%), followed by rivaroxaban, edoxaban, dabigatran, and warfarin (first-rank probabilities, 31.8, 15.9, 10.9, and 0.2%, respectively) (Figure 3A). In safety, apixaban ranked the best (first-rank probability, 71.4%), followed by edoxaban, dabigatran, warfarin, and rivaroxaban (first-rank probabilities, 21.0, 5.8, 0.9, and 0.8%, respectively) (Figure 3B).

**Table 3:** Rank probabilities of interventions on efficacy and safety.

**Rank probabilities**	**5th**	**4th**	**3rd**	**2nd**	**1st**
**Efficacy**					
Apixaban	0.0968187	0.1012725	0.1571750	0.2325012	**0.4122325**
Rivaroxaban	0.0433725	0.0966437	0.2026550	0.3377875	**0.3195412**
Edoxaban	0.1649275	0.1875575	0.2578213	0.2289262	**0.1607675**
Dabigatran	0.2123475	0.2640125	0.2442575	0.1739812	**0.1054013**
Warfarin	0.4825337	0.3505137	0.1380912	0.0268037	**0.0020575**
**Safety**					
Apixaban	0.0262600	0.0395525	0.0512362	0.1715538	**0.7113975**
Edoxaban	0.0582387	0.0943775	0.1248450	0.5095350	**0.2130037**
Dabigatran	0.1773537	0.3324900	0.2649787	0.1668375	**0.0583400**
Warfarin	0.0368625	0.3531388	0.4838112	0.1169038	**0.0092837**
Rivaroxaban	0.7012850	0.1804412	0.0751287	0.0351700	**0.0079750**

*The rank probabilities were used to reflect the hierarchy of drugs, and a larger first-rank probability value symbolized that the drug is more likely to be the best. The results of first-rank probabilities are shown in bold*.

Before initializing the Bayesian random-effect model, 1,000 adapting iterations were implemented to eliminate the effect of the initial value and obtain satisfactory convergence, and the results are shown in the trace plots and Gelman plots (Supplementary S3A,B, S4A,B). The heterogeneity of the study was analyzed by using the “anohe” approach, and the results showed that the heterogeneity of the study was low (*I*^2^ = 26%). The results of funnel plots (Supplementary S5A,B) and Egger's tests (*P* = 0.327 for efficacy outcome; *P* = 0.248 for safety outcome) showed that there was no obvious publication bias in the current study. In addition, as a reference, the results of the fixed-effect model of this study are shown in Supplementary S6. Due to the limited sample size of our study, we did not conduct any sensitive analysis or scenario analysis. Definitions of efficacy and safety outcome in the five included trials are presented in Supplementary S7.

## Discussion

The purpose of this network meta-analysis was to find the optimal drug for stroke/systemic embolism prevention in elderly patients with AF. In the current study, we found that DOACs were associated with a better effect on prevention of stroke/systemic embolism than warfarin, with apixaban being the best, followed by rivaroxaban, edoxaban, dabigatran, and warfarin. Regarding the safety of the drugs, apixaban was the best treatment too, followed by edoxaban, dabigatran, warfarin, and rivaroxaban. In conclusion, we found that apixaban was more effective and safer than any other DOACs and warfarin in this study.

Research indicated that in Australia, North America, and Western Europe, >70% of patients with AF are aged >65 years (33). Elderly patients with AF extremely needed anticoagulant therapy due to the their more serious symptoms than younger patients (6). Generally, elderly patients with AF have a higher risk of stroke/systemic embolism due to associated comorbidities such as hypertension, hyperlipidemia, diabetes, and chronic kidney disease (34). Thus, anticoagulation therapy was crucial for elderly patients with AF. However, they also have a higher risk of major bleeding in antithrombotic prophylaxis because of polypharmacy, decreased cognitive function, trauma, arteriosclerosis, and other bleeding risk factors (34, 35). Of elderly patients who started on oral anticoagulants, ~26% discontinue their medication within the first year. Of the elderly patients who discontinue anticoagulation, 81% cited safety concerns as the main reason for discontinuation (36). Therefore, it is critical to prevent elderly patients from suffering major bleeding or other side effects, for promoting the efficacy and compliance of anticoagulation.

In clinical trials, a left atrial appendage occlusion device (WATCHMAN device) has been proven to reduce the risk of major bleeding compared to single warfarin therapy (37). However, this kind of device requires continuous aspirin (one of the traditional drugs for stroke prevention) medication, which has an equivalent risk of major bleeding compared to warfarin (38). In contrast, DOACs were shown to be associated with a lower risk of major hemorrhage than warfarin in trials (10–13). Since the physical condition of elderly patients is weaker than that of young patients, using multiple therapies at the same time will definitely increase the pain of elderly patients. Obviously, the use of DOACs could replace the simultaneous use of WATCHMAN device and aspirin, which would undoubtedly improve patients' compliance with anticoagulation and reduce pain. Moreover, anticoagulation with warfarin required monitoring INR (indicator of human coagulation function) of changes every few days (39), while DOACs did not. Because of better convenience and compliance, DOACs could replace warfarin for stroke prevention and rhythm control interventions, including electrical cardioversion (40) and radio frequency catheter ablation (41), especially in elderly patients.

Although DOACs are known to be better than traditional drugs or the WATCHMAN device on antithrombotic prophylaxis, the efficacy and safety of these drugs are different from each other. Several studies had ranked each main DOAC (apixaban, rivaroxaban, edoxaban, dabigatran, and warfarin) in patients with AF (17, 42), but never had any research ranked each of them specifically in the elderly, which is the most important purpose of the current study.

After conducting network meta-analyses, for the first time, we found that apixaban ranked the best in both efficacy and safety for elderly patients with AF, especially in safety. Some real-world setting studies, systematic reviews, head-to-head clinical trials, and meta-analyses had recommended apixaban as the most effective and safe drug for anticoagulation, which was similar to our findings (19, 43–46). Warfarin is known to increase vascular calcification, suggesting increased cardiovascular disease events. Apixaban, which is an oral direct factor Xa inhibitor, had an exquisite ability in stabilizing coronary atherosclerosis and a strong inhibitory impact on prothrombin production. It is its good performance in pharmacokinetics and pharmacodynamics that makes apixaban superior to any other DOACs and warfarin (47, 48). Compared to apixaban, dabigatran had a higher rate of extracranial bleeding in anticoagulation (6). Furthermore, dabigatran should not be used by patients with a mechanical heart valve for antithrombotic prophylaxis (49). Several reviews and retrospective cohort studies indicated that apixaban has better effectiveness than edoxaban, rivaroxaban, and dabigatran for its shorter time to peak level and its longer half-life (6, 50, 51). Moreover, studies showed that apixaban was more cost-effective than warfarin, dabigatran, edoxaban, and rivaroxaban for stroke prevention in patients with AF (17, 52). Hence, clinicians who are going to prescribe DOACs for elderly patients in antithrombotic prophylaxis should firstly take apixaban into account.

Although apixaban has been proven to have the best safety in DOACs at this stage, hemorrhage is still its biggest side effect. Therefore, several new DOACs were created for better efficacy and safety in antithrombotic prophylaxis, such as betrixaban. Compared to other DOACs, betrixaban has a longer half-life, smaller peak–trough variance, minimal renal clearance, and minimal hepatic cytochrome P (CYP) metabolism, but it still needs more clinical trials to prove its priority (53, 54). Due to the lack of evidence for its efficacy and safety, we did not include betrixaban in the current study for analysis. We will continue to focus relevant studies on betrixaban in the future.

Network meta-analysis is an indirect comparative meta-analysis that refers to the indirect derivation of the relative effect of A vs. B through the interventions A vs. C and the intervention B vs. C. At present, there are two reasons for the use of indirect comparison in meta-analysis: (1) There are no original studies that involved direct comparison and (2) there are original studies with direct comparison, but their number is small or they are of low quality. Our study belongs to the first case described above, aiming to obtain the hierarchy among DOAC drugs by combining the results of RCTs on the comparison between each DOAC drug and warfarin. Since non-randomized studies may include selection, performance, and detection bias, we did not pool them together and included only RCTs or their reported data for better research validity. Network meta-analysis can evaluate multiple drugs simultaneously and find their hierarchy, to provide reference for clinicians in making the best choice for patients.

The five studies included in our research are all phase III RCTs, the participants involved in this network meta-analysis are all elderly patients (aged ≥75 years) with non-valvular AF, and the medication regimens in the control group are consistent across studies (28–32). However, there are some differences in patient baseline characteristics (mean age and the percentage of males), the duration of follow-up, and the number of patients lost follow-up, which may slightly impact the comparability of the reported data. For example, one study pointed out that the women treated with DOACs had a lower rate of major bleeding and a higher rate of stroke and systemic embolism compared with men (55). In the current study, a difference in gender ratios among the five included studies may lead to a slight reduction in comparability. But this difference is not very large, whether it will have a great impact on our network meta-analysis still needs exploration and so will other baseline characteristics.

Our study had a few potential limitations. First, the number of trials included in our study is relatively small. We will make further searching and investigation to include more trials and their corresponding reported data in our future studies. Second, due to the limited data, we failed to do a more detailed subgroup analysis in patients over 75 years old. Third, although the heterogeneity of network meta-analysis is low in this study, the power of network meta-analysis is relatively low due to the sample size. Fourth, the evidence network plot of this study is star shaped but not a closed loop, which indicates that there is a lack of head-to-head comparison data within DOACs; thus, we find no statistically significant differences between all comparisons in terms of efficacy or safety. Therefore, more RCTs that conduct head-to-head comparison within DOACs need to be implemented to achieve more robust results. Nonetheless, all the articles that we included are phase III RCTs with common control. They have the same dosing regimen of control and similar baseline characteristics, which reflected a high consistency among their study designs. Our study could still provide suggestions for the use of DOACs in elderly patients with clinicians. We will continue our researches in the future for a more complete conclusion.

## Conclusion

For the first time, our study demonstrated that in elderly patients with AF, DOACs show lower incidences of stroke/systemic embolism and major bleeding than warfarin, with apixaban being the best of those interventions. Therefore, apixaban should be given priority as an anticoagulant in stroke prevention for elderly patients with AF.

## Data Availability Statement

The datasets generated for this study are available on request to the corresponding author.

## Author Contributions

KD, JC, and SR performed the network meta-analysis and prepared the manuscript. HX and LL were responsible for the statistical analysis. YG provided editing assistance. All authors have received and agreed to this information before submission.

### Conflict of Interest

The authors declare that the research was conducted in the absence of any commercial or financial relationships that could be construed as a potential conflict of interest.
