# Supplementary material for: Efficacy and Safety of Direct Oral Anticoagulants in Elderly Patients With Atrial Fibrillation: A Network Meta-Analysis
Source: Front Med (Lausanne). 2020 Apr 7;7:107. doi: 10.3389/fmed.2020.00107 (PMC7154089; doi:10.3389/fmed.2020.00107)
Supplement: Supplementary file 1 [file Data_Sheet_1.docx]

Supplementary Material

**List of Supplementary**

S1: Table 1. Detailed search strategies for literature search on Aug.2019

**S2: Table 2. Quality of the included RCTs assessed by Cochrane risk of bias assessment**

**S3: Trace plots of the model for efficacy and safety outcomes**

**S3A:Trace plots of the model for efficacy outcome**

**S3B:Trace plots of the model for safety outcome**

**S4:Gelman plots of the model for efficacy and safety outcomes**

**S4A:Gelman plots of the model for efficacy outcome**

**S4B:Gelman plots of the model for safety outcome**

**S5:Funnel plots of the efficacy and safety outcomes**

**S5A:Funnel plots of the efficacy outcome**

**S5B:Funnel plots of the safety outcome**

**S6: Table 3.The results of fixed effect model on efficacy and safety outcomes ( lower triangle: stroke/systemic embolism; upper triangle: major bleeding)**

**S7: Table 4. Definitions of safety and efficacy endpoints in the 5 included RCTs**

| **Database** | **Search strategy** |
| --- | --- |
| **The Cochrane Library (Central)** | #1 (advanced age) OR (elderly patients) OR (older age)  #2 (Atrial fibrillation) OR (AF) OR (non-valvular AF)  #3 (anticoagulation) OR (antithrombotic) OR (anticoagulants) OR (warfarin) OR (dabigatran) OR (apixaban) OR (rivaroxaban) OR (edoxaban)  #4 #1 and #2 and #3 |
| **EMBASE** | #1 (Atrial fibrillation) OR (AF) OR (non-valvular AF)  #2 (advanced age) OR (elderly patients) OR (older age)  #3 (anticoagulation) OR (antithrombotic) OR (anticoagulants) OR (warfarin) OR (dabigatran) OR (apixaban) OR (rivaroxaban) OR (edoxaban)  #4 #1 and #2and #3 |
| **PubMed** | #1 (Atrial fibrillation) OR (AF) OR (non-valvular AF)  #2 (advanced age) OR (elderly patients) OR (older age)  #3 (anticoagulation) OR (antithrombotic) OR (anticoagulants) OR (warfarin) OR (dabigatran) OR (apixaban) OR (rivaroxaban) OR (edoxaban)  #4 #1 and #2and #3 |

**S1:Table1.** Detailed search strategies for literature search on Aug.2019

| Quality assessment criteria | ARISTOTLE,2014 | RE-LY,2011 | ROCKET AF,2014 | ENGAGE AF-TIMI48,2016 | J-ROCKET AF,2014 |
| --- | --- | --- | --- | --- | --- |
| Random sequence generation | **+** | **+** | **+** | **+** | **+** |
| Allocation concealment | **+** | **+** | **+** | **+** | **+** |
| Blinding participants and personnel | **+** | **+** | **+** | **+** | **+** |
| Blinding of outcome assessment | **+** | **+** | **+** | **+** | **+** |
| Incomplete outcome data | **+** | **+** | **+** | **+** | **+** |
| Selective reporting | **+** | **+** | **+** | **+** | **+** |
| Other bias | **+** | **+** | **+** | **+** | **+** |
| Overall quality score (maximum=7) | 7 | 7 | 7 | 7 | 7 |

**S2: Table 2.**Quality of the included RCTs assessed by Cochrane risk of bias assessment


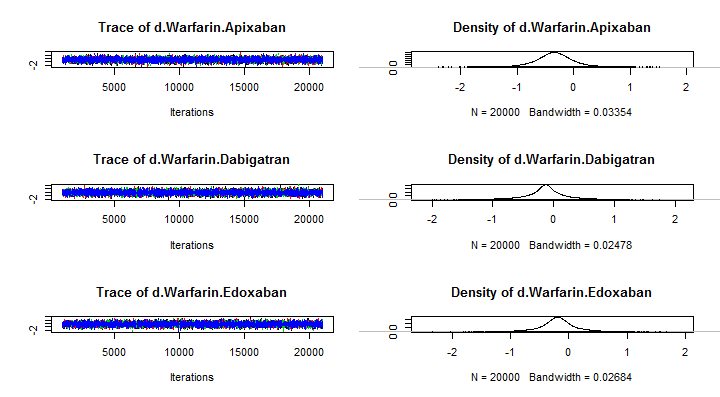

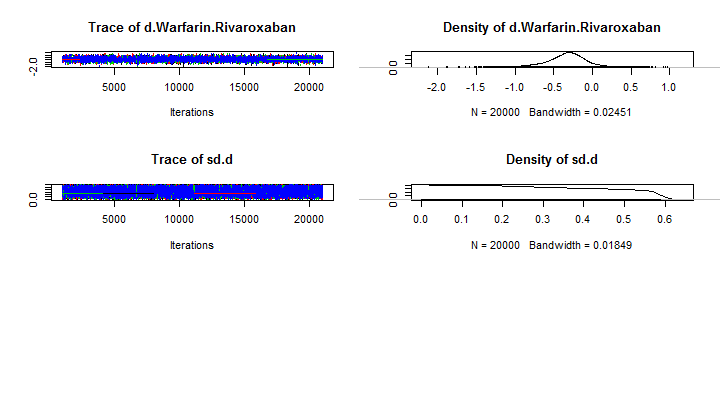


**S3A. Trace plots of model for efficacy outcome**


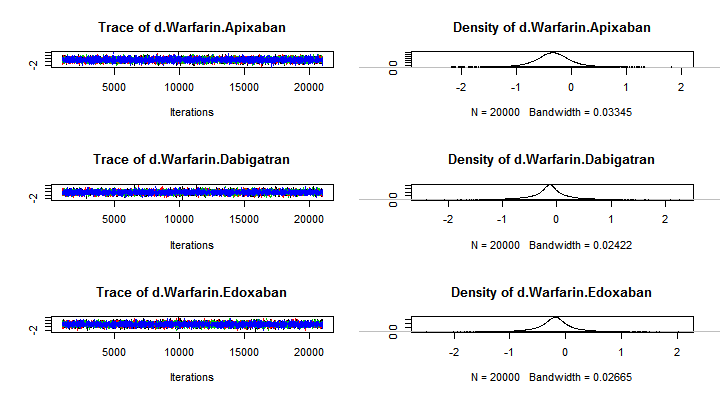

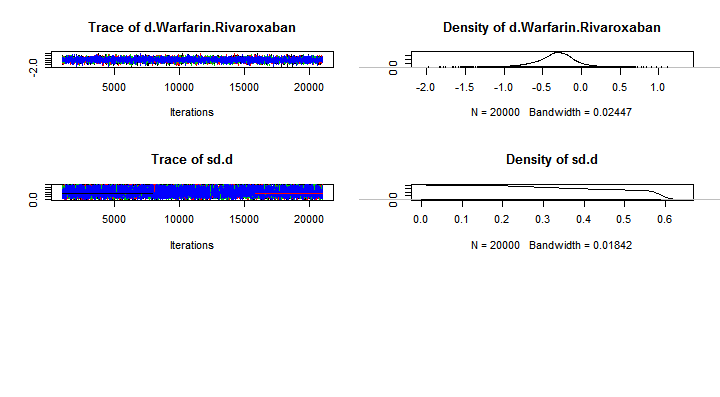


**S3B. Trace plots of model for safety outcome**


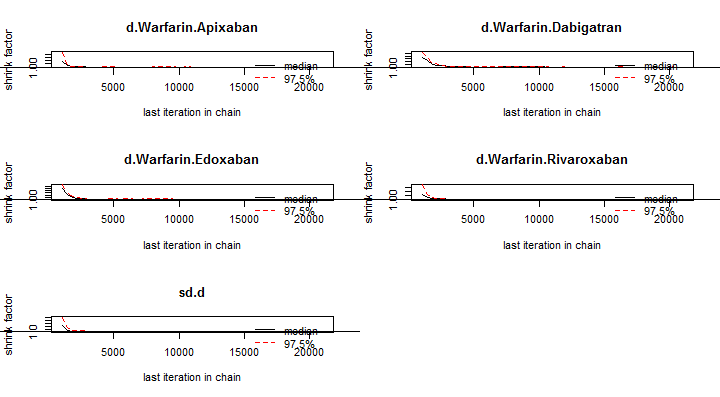


**S4A. Gelman plots of model for efficacy outcome**


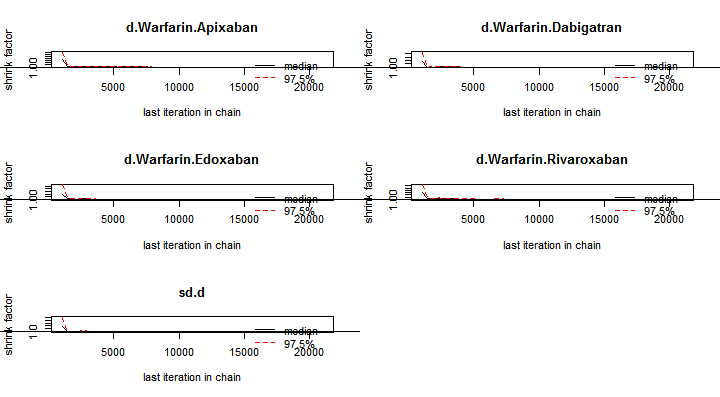


**S4B. Gelman plots of model for safety outcome**


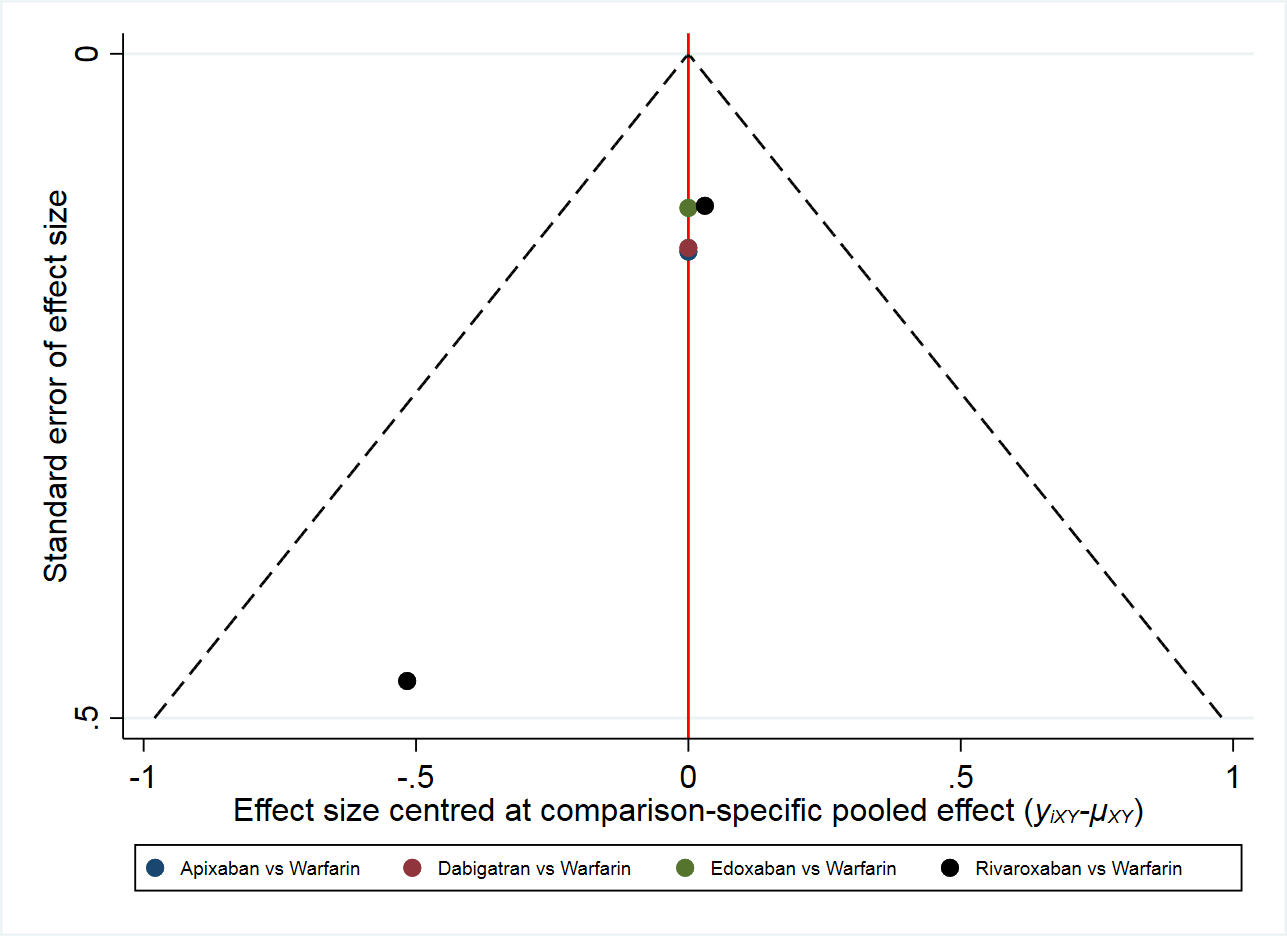


**S5A:Funnel plot of efficacy outcome**


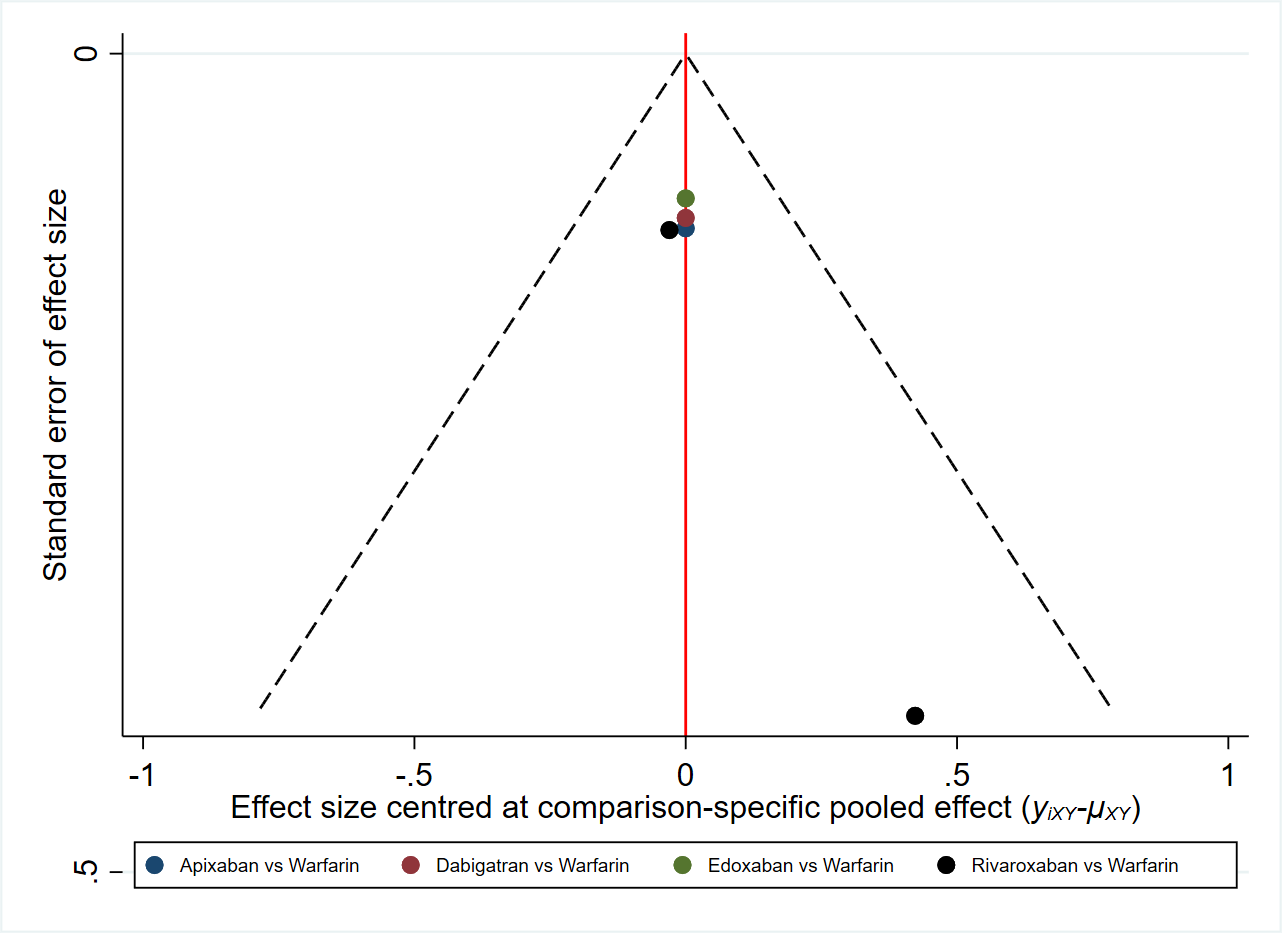


**S5B:Funnel plot of safety outcome**

| HR 95% CI | Warfarin | Dabigatran | Apixaban | Rivaroxaban | Edoxaban |
| --- | --- | --- | --- | --- | --- |
| Warfarin |  | 1.00(1.00,1.00) | 0.64(0.41,1.00) | 1.10(1.00,1.30) | 0.83(0.69,1.00) |
| Dabigatran | 0.88(0.77,1.00) |  | 0.64(0.41,1.00) | 1.10(1.00,1.20) | 0.83(0.68,1.00) |
| Apixaban | 0.71(0.50,1.00) | 0.81(0.56,1.20) |  | 1.80(1.10,2.80) | 1.20(0.81,1.30) |
| Rivaroxaban | 0.73(0.62,0.95) | 0.87(0.68,1.10) | 1.10(0.72,1,60) |  | 0.74(0.80,1.98) |
| Edoxaban | 0.83(0.68,1.00) | 0.94(0.75,1.20) | 1.20(0.78,1.70) | 1.10(0.81,1.40) |  |

**S6. Table 3 The results of fixed effect model on efficacy and safety outcomes ( lower triangle: stroke/systemic embolism; upper triangle: major bleeding)**

On the lower triangle, the column-defining treatment is compared to the row-defining treatment, and the hazard ratios (HR)<1 favors the column-defining treatment.On the upper triangle, the row-defining treatment is compared to the column-defining treatment, and (HR)<1 favors the row-defining treatment.

| **RCTs**  **(study name)** | **Stroke or systemic embolism** | **Major bleeding** |
| --- | --- | --- |
| **ARISTOTLE,2014** | Stroke or  systemic embolism | ISTH |
| **RE-LY,2011** | Stroke or  systemic embolism | ISTH |
| **ROCKET AF,2014** | Stroke or  systemic embolism | ISTH |
| **ENGAGE AF-TIMI48,2016** | Stroke or  systemic embolism | ISTH |
| **J-ROCKET AF,2014** | Stroke or  systemic embolism | ISTH |

**S7 Table 4.** **Definitions of safety and efficacy endpoints in the 5 included studies**
